# Therapeutic communication laboratory: integrating mixed methods with digital tools and reflective professional practice

**DOI:** 10.3389/fpsyg.2025.1447587

**Published:** 2025-03-12

**Authors:** Francisco Molinero, Gudberg K. Jonsson, M. Teresa Anguera, Laszlo Hunyadi, István Szekrényes

**Affiliations:** ^1^Mimesis, Institute for Psychosocial Studies and Analysis of Therapeutic Communication, Barcelona, Spain; ^2^Social Science Research Institute and Human Behavior Laboratory, University of Iceland, Reykjavík, Iceland; ^3^Faculty of Psychology, Institute of Neurosciences, University of Barcelona, Barcelona, Spain; ^4^Language Technology Research Group, Hungarian Research Centre for Linguistics, Budapest, Hungary; ^5^Institute of Philosophy, Faculty of Humanities, University of Debrecen, Debrecen, Hungary; ^6^Department of Digital Humanities, Faculty of Humanities, Eötvös Lóránd University, Budapest, Hungary

**Keywords:** therapeutic communication laboratory, conversational analysis in psychotherapy, therapeutic communication, analysis of temporal patterns, mixed methods, observational methodology, motivational interviewing, discourse of change

## Abstract

Communication has been recognized as the matrix in which the helping professionals operate in order to generate changes. In particular, psychotherapy is fundamentally a professional practice based, from its origins, on conversation. This study aims to establish the methodological and instrumental foundations of a therapeutic communication laboratory utilizing a mixed-methods approach. The primary objective is to develop and implement tools and procedures for the effective analysis of psychotherapy session recordings. We present a methodological proposal containing the foundations of a therapeutic communication laboratory which allows a new dialogue between professional practice and empirical research based on observation. We describe the processes of data incorporation and analysis of recorded sessions from a mixed methods perspective (Qual-Quan-Qual). The automation and integration of various digital tools, aligned with this mixed methods approach, are essential to achieve closer collaboration between practitioners and researchers. The incorporation of the transcription and labeling processes of the recordings into analysis tools such as Elan and Theme allows us to advance in these objectives. This approach enhances the training and supervision of psychotherapy professionals and bridges the gap between theoretical intervention models and their practical application. From a research standpoint, it enables the development of a knowledge base and observational instruments to advance the creation of more effective intervention models.

## 1 Introduction

The first studies applied to the analysis of the process of therapeutic communication had a marked qualitative character (Ruesch and Bateson, 1951), but if we take a broad perspective of the technological context of the evolution of knowledge in the field, we could observe that technological limitations have played a major role in its advancement. In this paper we want to highlight the opportunities, and the necessary instruments needed for a therapeutic communication laboratory with the implementation of mixed methods, given the nature of the communicative behavior under study (Ruusuvuori, 2010) and how the digital tools that we currently have at our disposal make it possible.

The methodology of conversational analysis (Antaki, 2008) that some authors have proposed to use for the analysis of sessions in psychotherapy (Peräkylä et al., 2012a,b) is a predominantly qualitative methodological perspective They have highlighted the methodological need to label the different components that come together in the generation of effects of meaning in the conversation, which they wanted to collect in the complexity of the transcription systems used. Paralinguistic elements, silences, rhythm, and speaking turns are important from the start of the conversation. which attempted to record as many details as possible about the timing, tone, intensity, and pronunciation patterns as the conversational sequence unfolded, with speaking turns occurring one after the other.

In this methodological perspective, the adjacency pair (concatenated sequences of the interlocutors) of speaking turns has been the main focus of the analysis of the interlocutors' intervention sequences (Peräkylä, 2019). However, it is clear that additional sequencing patterns beyond the simple concatenation of the interventions must be used. Therefore, in our perspective of mixed methods, we will be able to identify other types of sequencing in the conversational process by using tools like Theme to analyze the temporal patterns of the interlocutors' interventions.

From the perspective of psychotherapy practitioners, a significant qualitative leap was achieved with work initiated at MRI and the so-called Palo Alto School in the latter half of the last century. Indeed, a new reflective practice in psychotherapy, based on the observation of communication occurring in sessions, has emerged since the 1960s, giving rise to systemic family therapies and brief therapies at the Palo Alto School.

It will be in the mythical MRI of Palo Alto that the work of anthropologists, linguists, psychiatrists, psychologists, social workers converge (Bateson and Winkin, 1990), all of them concerned with how efficiency could be promoted in their interventions, and for this they used mainly the Gessell one-way mirror to observe the equipment what was happening inside the room where the psychotherapy sessions were taking place (de Shazer, 1999).

In addition to providing a rich conceptual and practical perspective on how to intervene in the communication process in the most efficient way possible to achieve the desired results of change, this interdisciplinary approach focused on the communication processes that successfully generate (giving rise to the so-called brief therapy) change in patients (Watzlawick et al., 1974).

These contributions to the understanding and efficient intervention in generating change were based from the beginning on observation and listening devices to follow the sessions live. Since then, based on these observation processes of what happens in the psychotherapy room (de Shazer, 1999)^1^ intervention models and techniques have been proposed such as motivational interviewing (Miller and Rollnick, 2015) or solution-focused interviewing (De Jong and Berg, 2008), which are intervention models based on the communication process, and which have been developing different techniques to facilitate change processes. It is also worth highlighting also, the contributions of third generation therapies developed from contextual psychology and the theory of relational frameworks developed in an experimental setting (Törneke, 2017) have also proposed the centrality of language functioning in change processes (Villatte et al., 2016).

These methodological perspectives (conversational analysis) and intervention models based on the language of change and therapeutic communication (reflective practice of psychotherapy professionals based on the observation of what actually happens in the therapeutic conversation) require the incorporation of the technological infrastructure that facilitates the process of reflective practice of professionals, and at the same time, do so with a mixed methods perspective that can collect and take into account, in a systematic way, the multimodal (Stivers et al., 2011) and multidimensional complexity of the object of observation, that is, the communication through which a helping professional promotes processes of change.

## 2 Components of the therapeutic communication laboratory

The Therapeutic Communication Laboratory proposes to combine observational methodology, as a mixed method, with the methodology of reflective professional practice based on observation. Learning and improving professionally by observing your own practice or that of your colleagues requires the development of observational devices and instruments that facilitate reflection on what really happens in psychotherapy sessions. Currently there is a “gap” between practice and theory, between intervention objectives based on the intervention models followed by professionals and the ways in which they are applied in the conversation process.

In order to overcome this gap, a therapeutic communication laboratory has to facilitate, on the one hand, the process of observing and reviewing sessions, making it less costly in terms of professionals' time, and on the other hand, incorporate the most appropriate tools so that it can be done with a rigorous methodology adapted to the object of observation.

To make this approach possible between the needs of reflective professional practice, systematically incorporating the observation of what really happens in the interaction with clients, we have at our disposalthe automation of the transcription processes not only of the verbal content, but also of the automatic labeling tools for intonation (Szekrényes, 2015) or silences that are so important in different therapeutic processes.

For example, we know very well the importance of being able to establish a good therapeutic relationship and the generation of trust and security, etc. And we know that this occurs during the course of the conversation through the different dimensions of communication: the verbal content, that is, what is said, but also and fundamentally how it is said, that is, the intonation, the rhythm of speech, the gestures, the game of the gaze, and others. All of this forms a specific message that is transmitted in the conversational sequence and which are the components of the different conversational operations that the professional performs, whether questions, formulations, through which we carry out the different therapeutic tasks. All these aspects require a technological infrastructure that allows flexibility and ease of use by professionals, since they must be able to incorporate their own therapeutic objectives and notes in a qualitative way, but at the same time provide support to incorporate the multimodal nature and sequential of the conversation. On the other hand, this infrastructure requires the automation of annotation processes, whether of textual or phonetic transcriptions, as well as the rest of the dimensions of communication.

**Figure 1:**
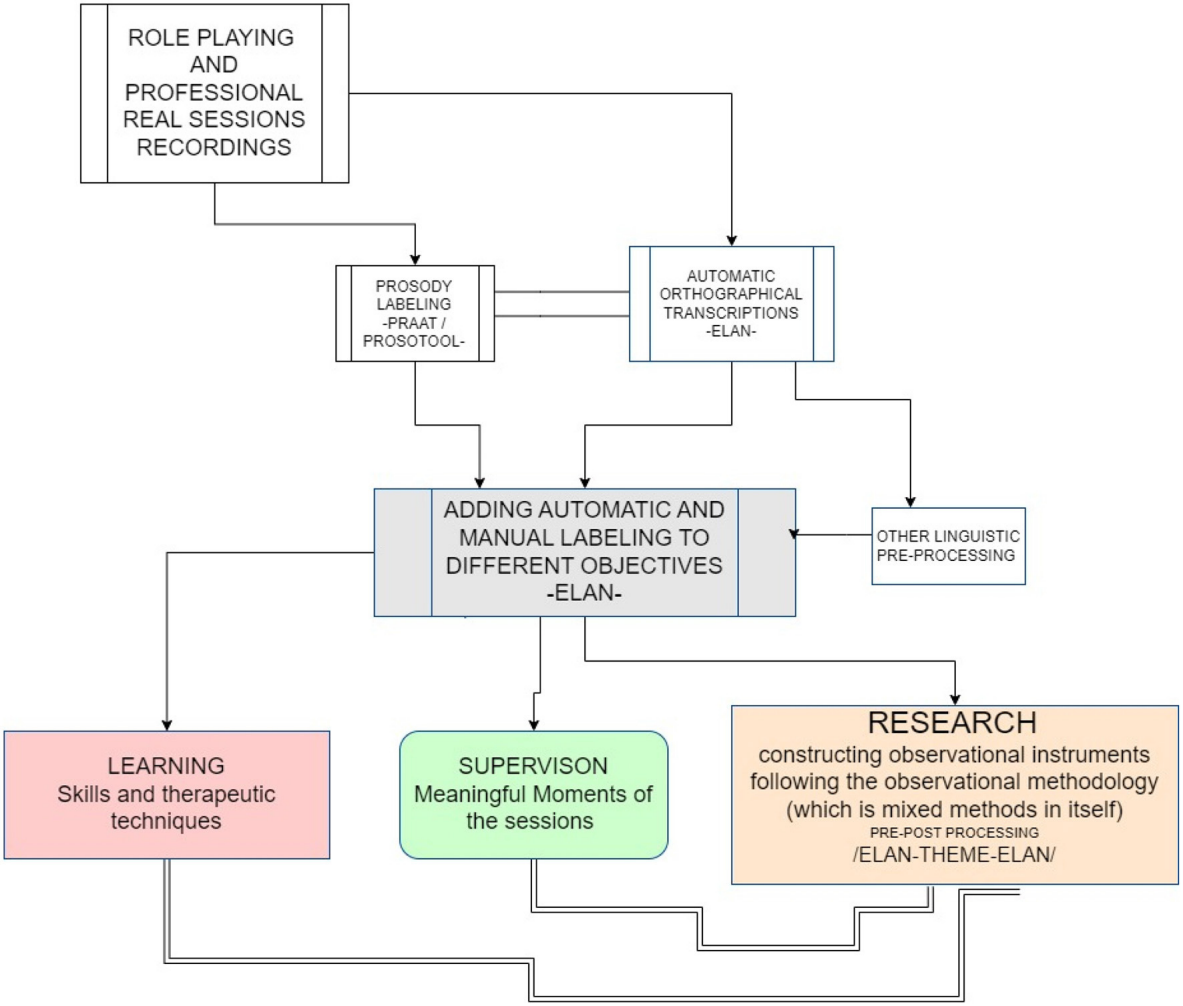
A knowledge base scheme made up of recordings from different contexts with different functions in the learning process and improvement in the use of different intervention models in psychotherapy.

As we can see in Figure 1, the transcription and labeling system combines, in ELAN, manual and automated annotation processes, allowing for great flexibility and facilitating moments of qualitative analysis and integration of automated processes that facilitate a review of the sessions with the least expenditure of time for professionals, while allowing their active participation in the development of annotation and coding systems adjusted to different objectives, whether in the field of supervision, training or research. On the other hand, it allows the development of observational instruments that can be used by researchers following mixed methods, Qual-Quan-Qual (Anguera, 2020), and the methodology of conversational analysis.

**Figure 2:**
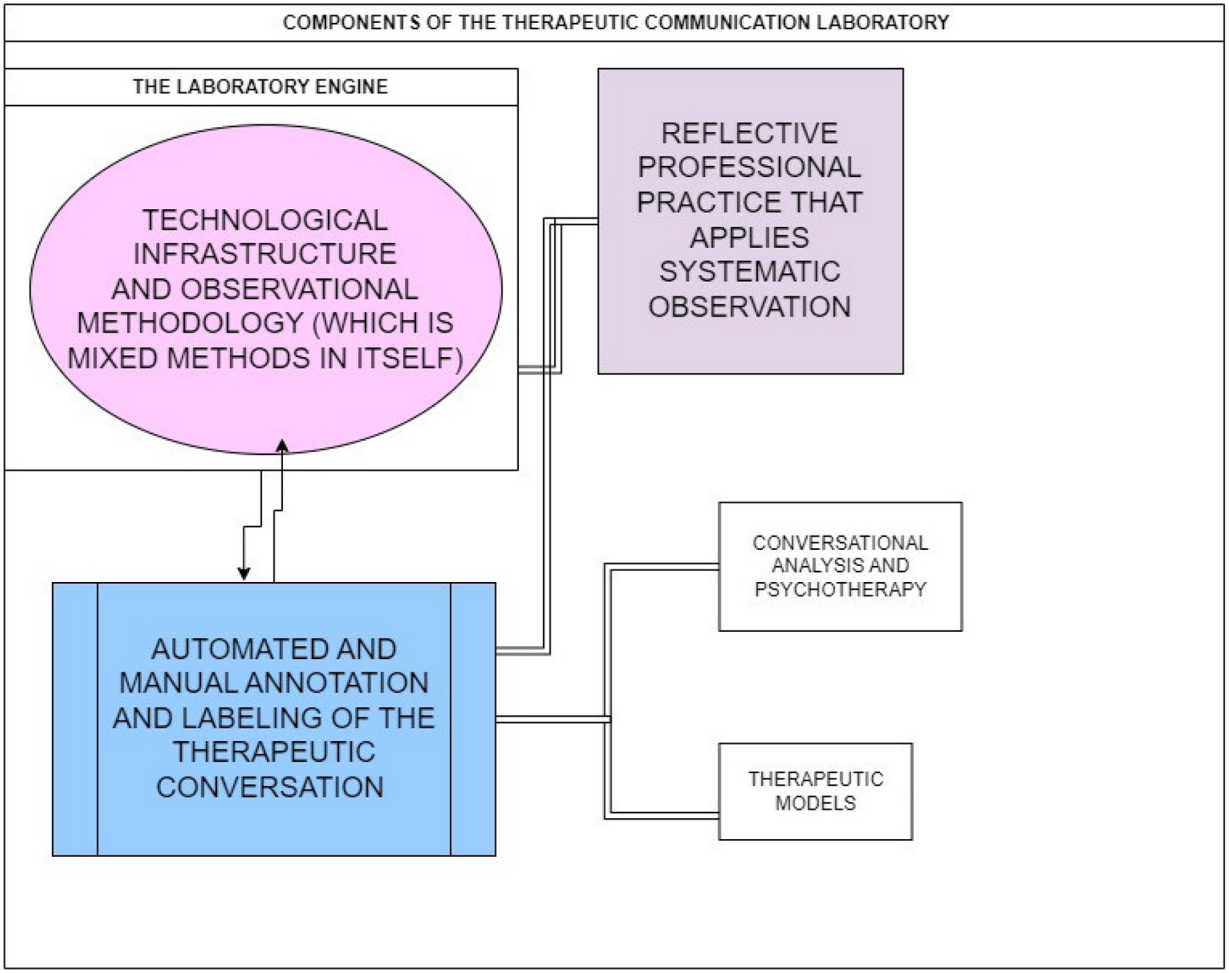
Three constitutive elements of a therapeutic communication laboratory.

As we can see in Figure 2, this technological infrastructure will make it possible to work toward overcoming the gap between professional practice and theoretical intervention models, since it facilitates the reflective practice of professionals and their incorporation into training, coaching, and supervision processes thanks to the flexibility and capacity to integrate different technologies from a mixed methods perspective.

## 3 Technological infrastructure on the analysis of therapeutic communication

Microanalytical lenses are the technologies that help us process the multidimensionality of communication in its temporal sequentially (Blanchet et al., 2005) and framed in the communicative actions oriented to change that the professional carries out in each case.

These “microscopes” and “X-ray” devices that allow us to X-ray communication are made up of computer programs and procedures for exchanging data analyzed in the light of these different digital tools. It will be on the basis of the creation of annotations and coding carried out with the help of automated labeling systems and complemented by qualitative and *ad hoc* annotations, that with the help of the exchange of these data with other types of temporal pattern processing, we will be able to build a knowledge base that supports learning and improving therapeutic communication skills and generating new knowledge in intervention models.

The different components pivot on ELAN^2^ (ELAN [Computer software], 2023). As explained on its website, ELAN is an annotation tool for audio and video recordings that allows you to add an unlimited number of textual annotations to audio and/or video recordings. This tool allows the import and export of different formats. Of particular interest to our mixed methods strategy are the exchange possibilities in the textgrid (Praat) formats that allow us, as we will see, to incorporate the results of automatic transcriptions, both orthographic and prosodic, and, on the other hand, the export of all the annotations aligned in time to the Theme program for analyzing temporal patterns.

In this way, Elan can be used both in a purely qualitative perspective by adding *ad hoc* in the review of role playing in learning processes, or the supervision of real sessions by facilitating the integration of automated processing of the recordings by adding a set of annotations resulting from different transcription procedures, as we will see, not only textual but also phonetic, while facilitating manual annotations by professionals with comments and identification of different, especially significant moments that occurred throughout the session.

It also provides a bridge that allows a fluid transfer between the world of practice (Bavelas, 2011) and that of research and the development of intervention models. through the development of systematic observation instruments to which temporal pattern analysis can be applied to identify statistically significant interaction sequences, true microanalytic lenses that allow us to observe how these patterns manifest by exploring them. again qualitatively. This knowledge base incorporates, through automated registration procedures, labels referring to intonation, along with transcription and other labels that can be added, as demonstrated with the use of the ELAN program, in order to integrate annotations referring to the different dimensions of communication (verbal, paralinguistic, and gestural).

On the other hand, we will see how to integrate transcription and tagging in ELAN with temporal pattern analysis programs (THEME)^3^ (Magnusson, 1996, 2000, 2020; Magnusson et al., 2016, 2023) that use algorithms to detect temporal patterns in those previously labeled files. They allow us to analyze the most significant patterns in which these labels appear organized and return to the qualitative analysis tools the representative sequences of certain temporal patterns to analyze in depth, generating a continuous movement of discovery, annotation and exploration of the different aspects that can be increase the effectiveness of professional intervention in these conversational sequences.

This back and forth movement between the use of ELAN, qualitative processing *ad hoc* based on the objectives of the annotations (learning contexts, supervisions, or for research purposes in which case, developing a systematized observation instrument that will depend on different objectives of exploration and analysis of the corpus, its analysis with Theme, and subsequently the possibility of returning to qualitatively analyze the temporal patterns detected, is at the heart of the mixed methods that we propose for the therapeutic communication laboratory.

There is consensus on the multimodal (Voutilainen et al., 2010) nature of communicative behavior—and specifically in therapeutic communication—, and it is highlighted in other sections of this article. The multidimensionality of communication has been studied by multiple classical and current authors (Bavelas et al., 2000; Hunyadi, 2020; Hunyadi and Szekrényes, 2020; Mondada, 2013), and this polyhedral character fits perfectly with a mixed methods approach to data collection, management and analysis.

We must take this multidimensional nature into consideration both from the perspective of qualitative analysis itself and from the creation of observational instruments that allow us a Qual-Quan-Qual integration.

## 4 Observational instruments and mixed methods

The exponential growth of mixed methods in the last quarter century has put the focus on the integration of qualitative and quantitative elements (Zhang, 2018, 2019; Zhang and Creswell, 2020), which has become the keystone on which discussions and publications continue, leaving behind complementarity as obsolete. Integration, considered as “the interaction or conversation between the qualitative and quantitative components of a study” (O'Cathain et al., 2007, p. 341) is a central axis around which most of the scientific production of recent years revolves (O'Cathain et al., 2007), and is also a key aspect in mixed methods research (Hitchcock and Onwuegbuzie, 2020, 2022). Moreover, there has been a very significant evolution from a dichotomous approach (qualitative vs. quantitative) to a *continuum* (Onwuegbuzie, 2012; Hitchcock and Onwuegbuzie, 2022), showing a wide arc of possibilities.

In previous works (Anguera et al., 2020; Arias-Pujol et al., 2022) we have referred to the connect way of integration, once proposed by Creswell and Plano Clark (2011), which has shown maximum efficacy to make effective the integration between qualitative and quantitative elements, through the three sequential QUAL-QUAN-QUAL stages, to which we will refer below.

We are interested in the analysis of therapeutic communication, which is part of the interactive habituality between therapist and patient throughout treatment. This aim justifies the suitability of observational methodology, a scientific method characterized by rigor and flexibility, and considered a *mixed method* in itself (Anguera et al., 2023a,b, 2017a,b), which explains its wide applicability in recent decades.

The study of therapeutic communication, characterized by the interest in analyzing the processes of change in the patient, must first address the delimitation of the spectrum of levels of response—in Weick's terms (1986)—, which have subsequently been called dimensions, and which in turn can be unfolded in potential sub-dimensions and in several levels. As specified earlier, therapeutic communication includes turns of speech with its verbal contents, with paralinguistic factors, silences, rhythm, ..., which gives it a polyhedral character. To organize the record, in the first QUAL stage, it is essential to build a customized observation instrument (Anguera et al., 2007), which is the “core” of this process, and which means much more than assigning labels.

First, a decision must be made regarding the dimensions to be considered, which must take into account both the theoretical framework and the situation under study (they can unfold in sub-dimensions of various levels). Together, the dimensions and subdimensions should form the “skeleton” or structure of the observation instrument and depending on the technology used in the recording (only tape recording or video recording) it will be possible to include only verbal behavior and part of the vocal behavior in the first case, or to open up all the possibilities of nonverbal behavior (Mondada, 2007) in the video recordings. The limits of quantification depends on the adequacy of observational instrument.

The second major initial decision is the segmentation of turns of speech into units, which is an important aspect that will later have an impact on the assignment of codes. Over the years, the location in the molecular vs. molar *continuum* of units has been a debated issue attending to the significance of the granularity to which we subject discourse sentences (Schegloff, 2000), and to the consequences of positioning. From the initial proposal of Krippendorff (2013), the approach we consider most appropriate (Anguera, 2021) is interlocutory segmentation (each turn of speech), and, within each turn of speech adopting the syntactic criterion (a grammatical sentence).

Once both decisions have been taken, and once the structure of the observation instrument and the segmentation criteria are available, it is time to proceed specifically to the development of the instrument. Based on each of the dimensions (or on the most molecularized subdimension, as the case may be), a catalog of behaviors (an open list of communicative behaviors that must be mutually exclusive) or, if possible, a system of categories (a closed list that must be exhaustive and mutually exclusive) will be drawn up. This customized observation instrument will be essential to transform the (qualitative) recording of turns of speech into a matrix of codes, which will also be qualitative in nature, but which will be systematized in such a way as to allow its quantitative analysis, thus making the integration of mixed methods a reality (Anguera et al., 2021).

The code matrix is irregular, and its structure will be subject to the syntactic rules of the program used to carry out the subsequent quantitative analysis.

The QUAN stage that follows the first QUAL stage has had to make its way. There is a conceptual plurality in mixed methods that can be evidenced from the words of Onwuegbuzie and Combs (2010), who state that “analyzing data in mixed methods is one of the most difficult steps—if not the most difficult step- of the mixed methods research process” (p. 397). In recent years, Onwuegbuzie and Johnson (2021) have developed the *crossover mixed analyses*, which imply that one or more types of analysis associated with one tradition (e.g., qualitative) can be used to analyze data associated with another tradition (e.g., quantitative). Among the multiple options presented in *crossover mixed analyses*, we highlight for its suitability the diachronic analysis—which is quantitative—from qualitative data—which we obtain in a systematic observation record—(Anguera et al., 2021).

In the framework of the diachronic analysis from qualitative data, and which allows us to materialize the QUAN stage, the quantitative analysis that we recommend here is the detection of T-Patterns (TPA) using the THEME program (Magnusson, 1996, 2000, 2020; Magnusson et al., 2016), which presents great possibilities of connection with the ELAN program (Szekrényes, 2019).

The T-Pattern is the core of the model, and its essence lies in the discovery of hidden structures, based on the critical interval between series of points with respect to the time dimension, revealing itself as an extremely valuable analytical tool, which in turn entails a permanent dialogue with the respective conceptual framework. Its non-visible character increases its power of discovery, since the researcher is interested in being able to extract the internal structure that shows the key to the behavior that occurs.

A T-Pattern is defined as the structure formed by a series of events that occur concurrently or sequentially more frequently than would be expected by chance if all the events were distributed independently. This series of events occur in the same order, maintaining temporal distances between them (critical interval) that are relatively invariant to the null hypothesis that each event is independent and randomly distributed at the temporal level (Magnusson, 1996, 2000).

A great advantage of T-Pattern detection is that it is not constrained by implicit assumptions about the distribution of the analyzed behaviors and allows the selection of minimum number of occurrences and significance level, among other parameters, with a clear control over chance findings.

THEME^TM^ (2008) software, throughout the development of its different versions, has allowed the detection of T-Patterns, and the wide applicability deployed has allowed showing self-similarities at different scales, from brain neuronal to social and group interaction.

In recent years, T-Patterns detection has been applied in observational studies in different domains, as shown by two recent systematic reviews (Anguera et al., 2023b; Sáiz-Manzanares et al., 2022), confirming its robustness.

## 5 Contributions of the HuComTech project to development of a knowledge base of therapeutic communication

As we have already mentioned, in a conversational sequence, verbal content is combined with paralinguistic intonational components and kinesic and gestural components. All of them behave like a pack of information that unfolds over time and form different temporal patterns that are responsible for the generation of sense, meaning and effectiveness of conversational interaction. Hence the need to incorporate into the analysis procedures of therapeutic actions a space-time mesh formed by those components of communication that make up the background in which the different therapeutic communicative actions make sense and operate.

The integration that we propose between observation and qualitative annotation and the eventual development of observational instruments, as described in the previous section is based on the creation of a corpus of therapeutic communication recordings created from different professional practices, namely, role playing in training workshops, real sessions collected for the supervision of psychotherapy professionals or other professional areas of communication that have the generation of change processes as a late motive. This corpus is processed based on different objectives, whether research or exemplification of different types of therapeutic skills, or reflection on how different therapeutic tasks are carried out.

This corpus, whether for a purely qualitative treatment, or for subjecting it to microanalytical analysis procedures through its integration with temporal pattern analysis tools following the perspective of mixed methods, can be transcribed and annotated, preferably automatically, such as we will specify in this paper. For this, automatic transcription and phonological analysis tools are essential to the extent that they save a large amount of manual transcription and annotation time, and therefore, their incorporation into professional practice contexts is much more feasible. On the other hand, this precision in the transcription and the levels of analysis taken into account in the review of the sessions and subsequent coding acquire greater quality and depth.

In the treatment of the corpus, we take as reference what was done by the HuComTech project (Hunyadi, 2011; Hunyadi et al., 2011) of which several of the signatories are authors, has had a pioneering role in the exploration of conversational corpora applying a multidimensional perspective. The multimodal nature of communication served as a research focus that resulted in the creation of the highly elaborate and multidimensional HuComTech. This corpus is based on about 30 hours of formal and informal dialogues with 200 participants with the aim of capturing, describing, and learning about those verbal and non-verbal properties of communication.

From the creation of this corpus, interesting contributions have been made on how we could investigate different aspects that are relevant to the purpose of a therapeutic communication laboratory. One of the aspects that we can explore with this methodology is how different conversational forms indicate different mental states. When engaged in a personal interaction, the interlocutors do not only convey factual information, they also communicate their attitudes toward both this information and its source, the partner of the interaction. Attitudes can vary from being social/psychological to pragmatic/situational, to active/reactive, and much more. Spoken interactions are not just sequences of grammatically well-formed utterances; personal real/time involvement in a dialogue is realized in a rich, multifaceted, multimodal (Voutilainen et al., 2014) environment with each modality contributing to the holistic nature of the resulting message. The temporally dynamic nature of dialogues means that interactions shape and change their course in real time, with the formal properties of the constituting modalities evolving in parallel with the cognitive processes behind them. Quite noticeably, the grammaticality of a verbal utterance cannot easily be judged following the rules applicable to a temporally static written text—due to their often high degree of fragmentation. Importantly though, we can consider them perfectly fitting the conversation, since this linguistically fragmented speech is just a single component of the multimodal complex.

By studying the linguistic fragmentation of a conversation one can add yet another layer to the understanding and interpretation of the interaction, often hidden behind the spoken word. This was the goal of a study aimed at identifying the patterns of spoken utterances within the HuComTech corpus as well (Hunyadi, 2013). Since automatic morphological and syntactic analysis is always language specific, the AI-based application *magyarlanc*, developed for Hungarian, the language of the corpus, was used for the specific purposes of this study.^4^ The output of the analysis offered a hierarchical syntactic representation of the actual utterance that also included of a number of structurally incomplete sentences. Such chunks of speech, often agrammatical from a strict theoretical nature can in turn shed light on the state of mind of the speaker, including, among others, their assertiveness, uncertainty, presence or lack of cooperativeness, all relative to the given conversation. The CMU Link Grammar^5^ natural language parser, together with its actively developed tool^6^ offers an easy to use platform for a basic syntactic analysis of sentences with an output showing existing as well as missing links within the sentence. These missing links can then serve as indications of structural incompleteness possibly associated with some developing state of mind.

These qualities can be supported by observing other layers of linguistic structure as well. One of the most outstanding of them is prosody. Prosody includes intonation, intensity and duration. The automatic annotation of intonation (cf. *ProsoTool* mentioned earlier) offers a stylized representation of the tunes spreading across the utterance which, if associated with word and/or sentence boundaries, can be an important source of information about the state of mind of the speaker. Automatic segmentation of speech into words (word alignment) is available^7^ for a number of languages after submitting an audio (or video) file together with its orthographic transcription. It has a variety of output formats, including “.eaf” (for ELAN) which can easily be imported and associated with other, already existing levels of annotation. Intensity data (yet another output of *ProsoTool*)^8^ add extra information to the way the given conversation is performed, shedding light on, among others, the expressed emotions manifested between the interlocutors. Duration data can also be quite informative: a short word followed by a longer pause can indicate hesitation or contemplation, especially following conjunctives, such as *if* , *and* or *but, however*, or between parts of a compound.

We understand the conversation process in its space-time deployment, forming a multidimensional mesh on which we must place the necessary lenses to be able to analyze how the different interventions and processes of change are taking place. These lenses are provided to us by these computer tools ELAN, PRAAT, THEME and very fundamentally by the developments of artificial intelligence for transcriptions and automatic labeling of paralinguistic dimensions (ProsoTool) (Szekrényes, 2015). Without these instruments it would be very difficult to observe the deployment and effects of professional interventions in the development of the sessions.

## 6 Motivational interviewing and the language of change

As an example to show the possible integrations of analysis instruments of psychotherapy sessions from this mixed-methods perspective, based on this technological infrastructure, we are using a publicly available recording of a motivational interview.

Motivational interviewing is an intervention model based on language and communication that has developed coding systems^9^ of the different skills that operate in the interview to evaluate the degree to which professionals apply the model.

Motivational interviewing is a collaborative, client-centered intervention model designed to explore and strengthen internal motivation for change. It has been used especially in the treatment of alcohol problems, but it has been extended to other areas of intervention, both psychotherapy and other contexts of professional intervention for the facilitation of change processes (health, educational, social or organizational contexts).

The interventions of the professional in the motivational interview are such as open questions, or formulations such as Reflections,^10^ Affirmations, or Summaries through which the person will be guided to generate for himself different forms of language of change aligned with the client values that lead you to commit to change based on your own intrinsic motivations.

It is assumed that the presence of different forms of change language in the person's speech that is manifested throughout the interview will lead him to take the necessary steps to make that change a reality in his life. Therefore, the interviewer's interventions will be aimed at eliciting that type of language in the person interviewed.

From the perspective of conversational analysis, in which we position ourselves, it is necessary to analyze therapeutic communication taking into account how speaking turns are concatenated, and therefore, analyze the interventions of both the professional and the client. In this sense, it is worth highlighting the contributions of linguists such as Laws et al. (2018), Apodaca and Longabaugh (2009), or Amrhein (2004), who have contributed to the motivational interview model with a classification of the different forms of language of change.

## 7 ELAN annotation and labeling program for the analysis of therapeutic communication

ELAN [Computer software] (2023) is an open source program created and maintained by the Max Planck Institute for Psycholinguistics, which allows us to link different types of annotations, transcriptions and labeling of the different dimensions of communication (paralinguistic or gestural) by linking them to the timeline of audio-visual recordings. As its creators say (ELAN [Computer software], 2023) “a user can add an unlimited number of textual annotations to audio and/or video recordings. An annotation can be a sentence, word or gloss, a comment, translation or a description of any feature observed in the media. Annotations can be created on multiple layers, called *tiers*. Tiers can be hierarchically interconnected. An annotation can either be time-aligned to the media or it can refer to other existing annotations.” (see text footnote ^2^).

On the other hand, it allows us to work either qualitatively, generating *ad hoc* labeling based on the specific objectives we have when viewing the sessions (for example, identifying moments of success, more significant interactions for learning, training or supervision tasks of professionals), but also develop a systematic coding system that allows us to feed a knowledge base that can be used in research or training Programs.

Likewise, it has a very interesting capacity for integration with other tools, especially with the PRAAT program for phonetic speech analysis, which, as we will see in this paper, is very useful in the process of automating transcription and annotations of the paralinguistic dimension and emotional prosody.

Along with these capabilities of integration and alignment in time of the different components to be taken into account in the analysis of therapeutic communication, it is also very suitable for integrating it into research processes with mixed methods and analysis of temporal patterns (Anguera et al., 2023a,b) following the methodology proposed by Hunyadi et al. (2016) and Szekrényes (2019) that allows us to carry out a round-trip movement of integration between the moment of annotation and qualitative analysis, and analysis of quantitative temporal patterns. For this reason, it is the most appropriate tool to label communication sessions because we can analyze how the different dimensions that make up communication unfold over time, that is, a space-time mesh formed by the words used, the tone, the rhythm, gestures,... and together with these dimensions unfolding in conversational sequences, we align the codings that correspond to the actions carried out by the therapist.

In this paper, our purpose is to show the usefulness and fit of the methodology and tools proposed for the analysis of intervention processes in psychotherapy. In this sense, in the case of motivational interviewing, the integration of the tools presented below allows the joint incorporation of the necessary requirements for methodological rigor and the facilitation of the work of professionals through automation of the processing of recordings and the possibility for them to incorporate their own reflections in future observation instruments.

**Figure 3:**
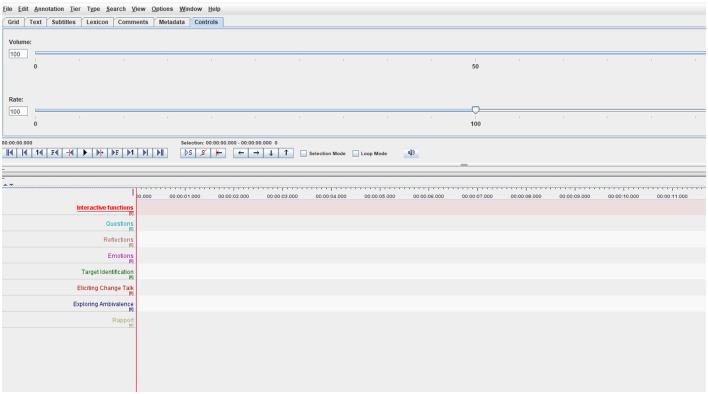
A knowledge base scheme made up of recordings from different contexts with different functions in the learning process and improvement in the use of different intervention models in psychotherapy.

In the Figure 3, we show how we can manually add the Tiers with therapist annotations depending on the intervention model or the specific objectives of the interventions. In the case of motivational interviewing, we can add where Reflections, open questions, or different modalities of change discourse have occurred. In other intervention models, such as EMDR therapy, we could note where references to Target Identification appear for processing.

## 8 Automated transcription and labeling of the motivational interview

**Figure 4:**
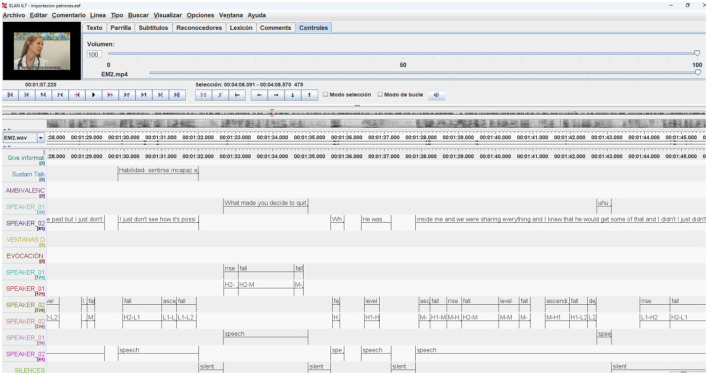
Time-aligned annotation tiers in ELAN. Video thumbnail from “The Effective Physician: Motivational Interviewing Demonstration”, YouTube, uploaded by MerloLab, Nov 26, 2009.

As we can see in Figure 4, the ELAN program allows us to label the different dimensions of communication, the verbal content of the transcription as well as integrate the automatic labeling carried out with Prosotool (Szekrényes, 2015), a method for automatic annotation of fundamental frequency, in the PRAAT program The exchange between ELAN and PRAAT is direct so we can work together with these two programs.

ProsoTool requires prior annotation of speaker changes to separately analyze participants' intonation. This step can be automated using available open-source speaker diarization tools like the Pyannote Python module (Plaquet and Bredin, 2023). The resulting speech segments, labeled per speaker, can then be utilized as a multi-tier representation of turn-taking for prosodic and T-pattern analysis. ProsoTool's output consists of categorical labels aligned with the audio, describing the shape (rising, falling, descending, ascending, level) of pitch curves as longer trends in the development of fundamental frequency values. These labels for intonation movements are complemented by positional ones that determine the location of the actual curve within the speaker's relative vocal range (Low1, Low2, Medium, High1, High2).

For automatic transcription, there are publicly available models from OpenAI's Whisper (Radford et al., 2022), known for its high accuracy with the Spanish language. The outputs of speaker diarization, speech recognition, and prosodic analysis can be effortlessly combined in Praat TextGrid format, then imported into ELAN as previously mentioned above.

To implement this processing chain, we utilized a Python-based system developed at the Department of Digital Humanities, Eötvös Loránd University. This system incorporates Pyannote (*Speaker-Diarization-3.1*)^11^ for speaker turn detection and the *openai/whisper-large-v3* model^12^ for speech recognition. Following post-processing, the results are provided in multiple formats, including TextGrid, which was used to input data into ProsoTool, an open-source tool available on GitHub.^13^

In the Figure 4 we can see how the different annotations aligned in time contain the different aspects of communication, the verbal content, the silences, the prosodic characteristics of the speech of both the therapist and the client, as well as other labels such as the client's expressions of change speech, of what type, as well as the type of intervention that the professional has carried out. All this information is aligned in time and can therefore be exported to a temporal pattern analysis program to identify the most significant patterns.

The automatic transcription procedures of verbal content and intonation will facilitate the process of qualitative exploration of the session. Therefore, in this first moment, we will be able to explore, analyze and label manually depending on the objectives and context of use (training, supervision or research) of the recording.

Likewise, we can also develop an observational instrument with a system of categories that collect the different dimensions of communication as well as the categories that we want to analyze, whether they refer, as in the case of the motivational interview that we take as an example, to the different types of client expressions, whether change speech or maintenance speech, and on the other hand, the different types of therapist interventions to determine possible temporal patterns with Theme.

For this, the “back and forth” movement between ELAN and the THEME program (Szekrényes, 2019) is of fundamental importance, since it allows not only to identify possible statistically significant temporal patterns, but also to subsequently analyze some of these patterns in a qualitative way in the ELAN program.

## 9 Analysis of temporal patterns with THEME

In order to develop a deeper understanding of therapeutic communication, one should start out from the linguistic form and continue to observe and understand the multimodal moments of the interaction, and, finally, go beyond both the verbal and nonverbal stereotypes and arrive at the patient as a single independent individual. It is, undoubtedly, not an easy task, and the therapist's observations and judgements have to be based on as solid data as possible. This is the point where a collection of data in the form of a comprehensive behavioral corpus can be of great importance.

The following three examples are taken from the HuComTech multimodal corpus, in order to demonstrate how a corpus of therapeutic communication will prove to be useful once it is completed. The patterns are all taken from dialogues of a more generic nature using Theme, the research environment especially designed to uncover even such patterns of behavior which are hidden from the naked eye. Cf. (1), a rather complex multimodal pattern consisting of both interpretive events (emotional, attitudinal and conversational) and descriptive ones (gazing and prosodic), with the bracketing representing the hierarchical structure of the pattern as a whole:

(1)       ([(( v emot,e,natural,moderate up agr,b,uncertainty )( mp sptopic,b,t init v gaze,b,left,down ))][( p spf0mov,b,fall p spintmov,b,stagnant )])

Following the bracketing, this pattern consists of two main sub-patterns, the first ending with v gaze,b,left,down. Each sub-pattern is binary, consisting of two event-types each. The meaning of (1) is the following:

The expression of a moderate, natural emotion on the part of the speaker (the interviewee) is associated with the expression of uncertainty of agreement (Sidnell, 2006); after that the speaker initiates a topic associated with their gaze pointing left and down. This whole complex of sub-patterns is then statistically the predictor of the following sub-pattern, i.e. the speaker's tone starts falling and then becomes stagnant. The total duration of the pattern is 5,526 s. The probability of it being formed by chance is *p* = 0.00000013, according to the binominal test embedded in the Theme search algorithm.

**Figure 5:**
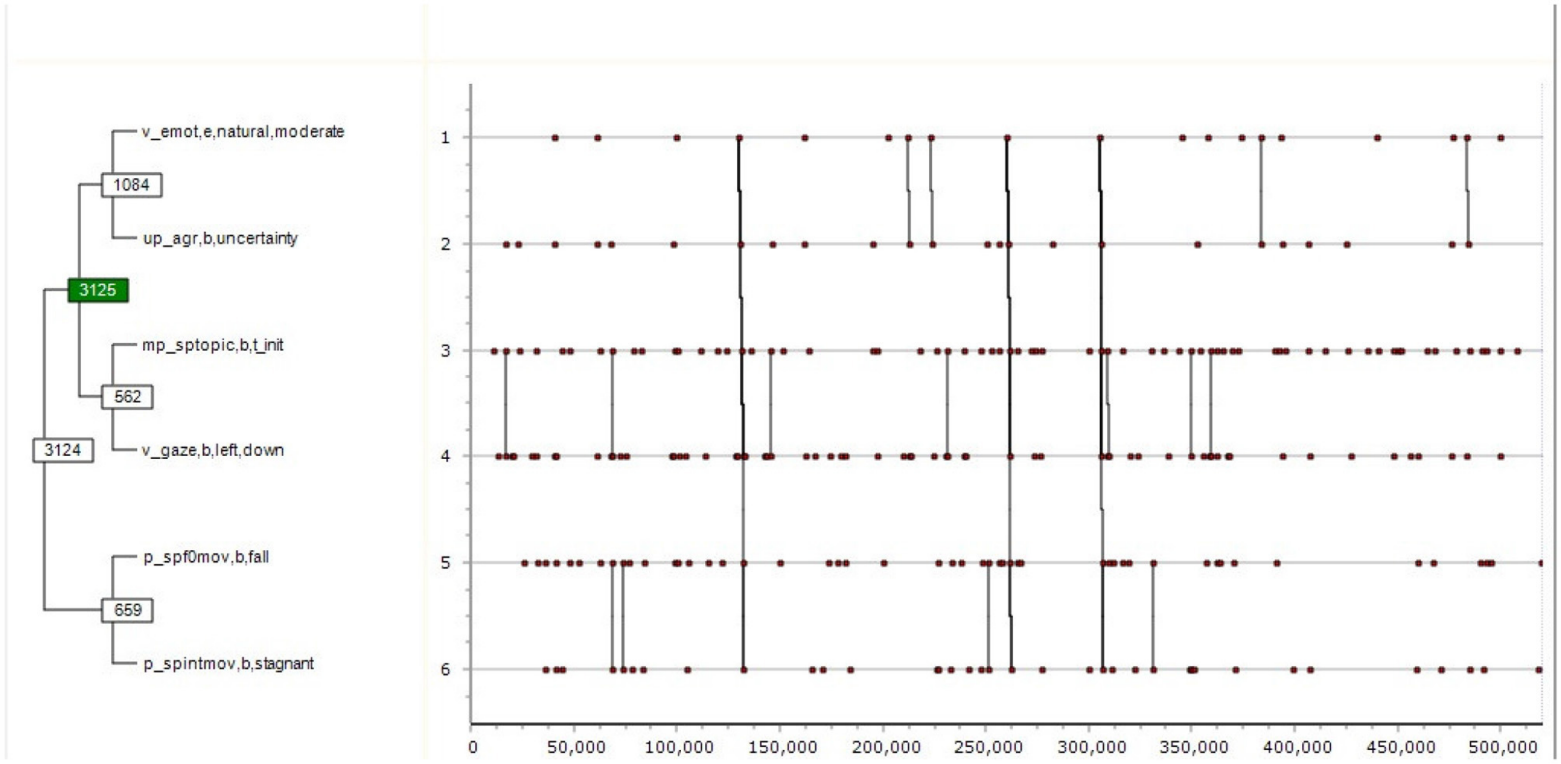
The tree-like form of T-patterns.

Figure 5 displays the above pattern in a tree-like form, where the immediate binary patterns are associated with a direct line. The vertical dimension of the figure follows the relative time of the pattern, while its horizontal dimension its absolute time. The numbers on these lines are the IDs technically standing for the given pattern within Theme.

(2)       ([(( v gaze,b,blink v post,e,upright )( v post,b,lean,back mp sptopic,b,t change ))][(( v gaze,e,blink v post,e,lean,back )( v post,b,upright mp sptopic,e,t change ))])

**Figure 6:**
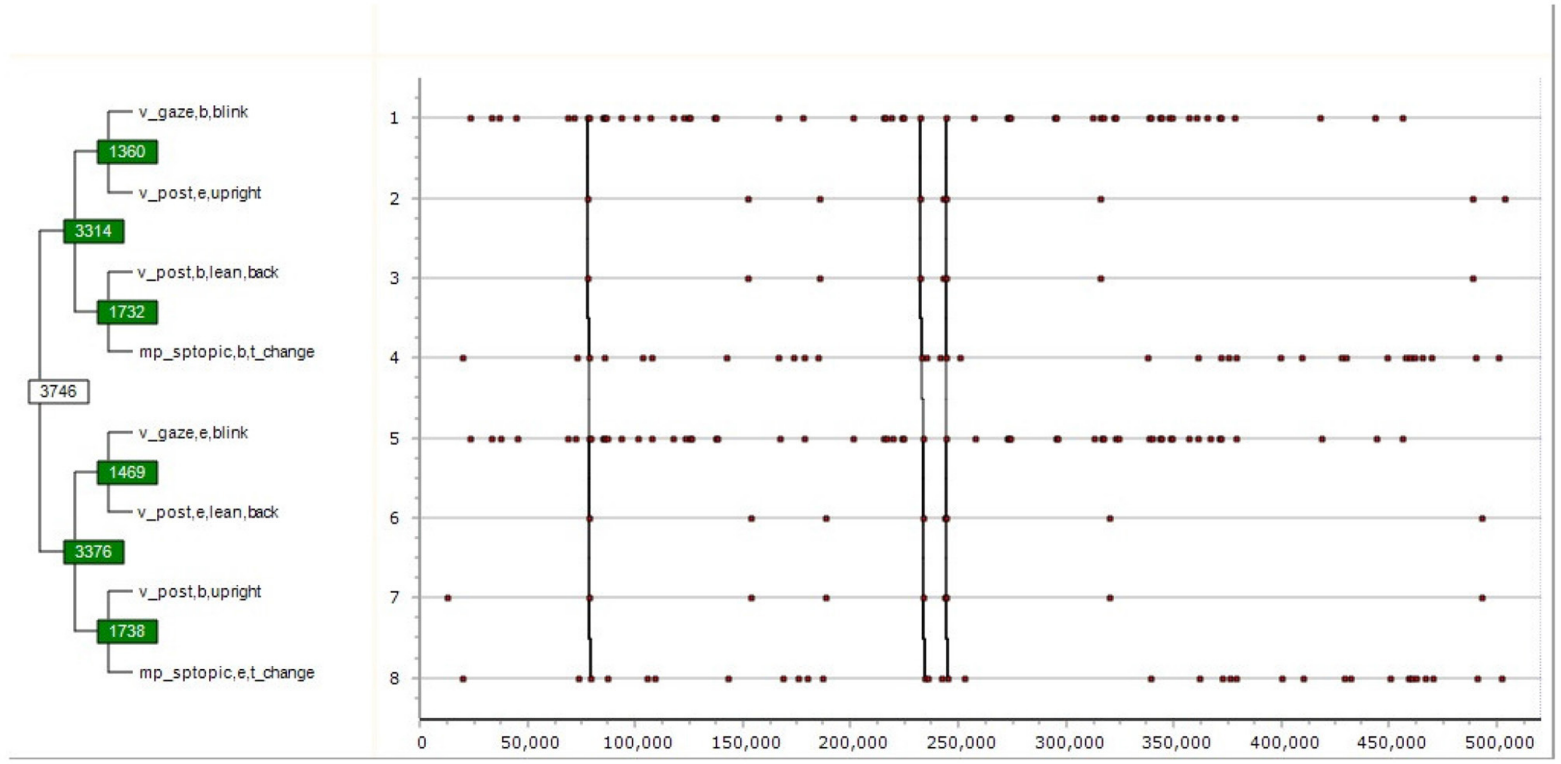
T-pattern followed by a sub-pattern.

This pattern also consists of two main sub-patterns, the first ending in mp sptopic,b,t change. The meaning of (2) is the following:

The speaker blinks and their posture ends in upright position; the they start leaning back while starting changing topic. It is followed by another blink and they lean back; followed by yet another sub-pattern: moving upright while finishing the topic change. The total duration of the pattern is 4,403 s. The probability of it being formed by chance is *p* = 0.00000075. See also Figure 6.

(3)       ((( up att,e,paying up agr,b,uncertainty )( v gaze,b,left,down v gaze,e,left,down ))(( mp sptopic,b,t elab mp sptopic,e,t init )( p spintmov,e,stagnant mp sptopic,e,t elab )))

This pattern, also consists of two main sub-patterns, the first ending in v gaze,e,left,down. The meaning of (3) is the following:

The speaker finishes paying attention and expresses uncertainty of agreement, starts and finishes directing their gaze left and down; then the speaker starts elaborating a topic while finishing initiating it, to be associated with the ending of a stagnant intonation while finishing the topic-elaboration.

**Figure 7:**
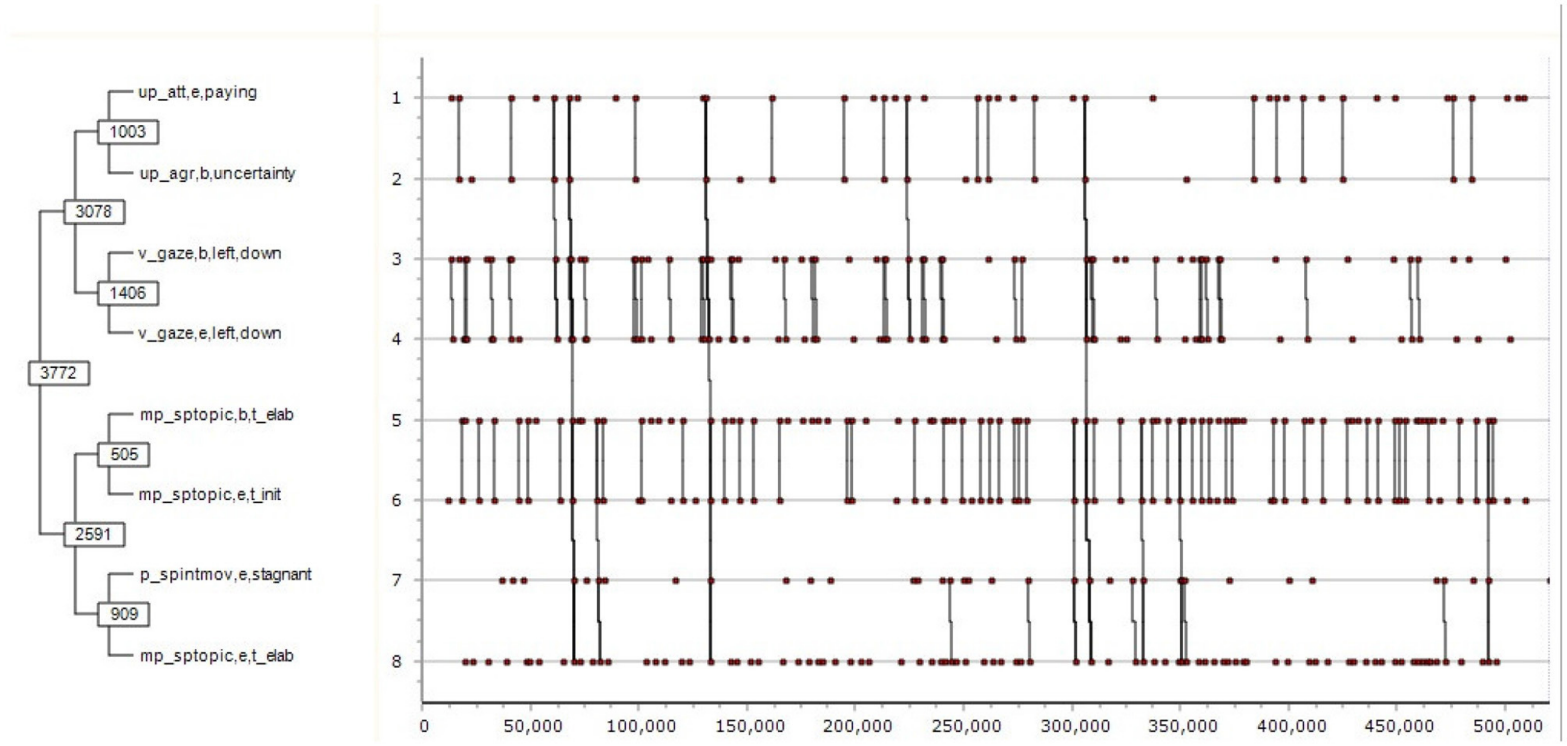
T-pattern containing two main sub-pattern.

The total duration of the pattern is 7,043 s. The probability of it being formed by chance is *p* = 0.00001892. See also Figure 7.

In its process of pattern discovery, based on a predefined “critical interval” Theme first associates two events into pattern1 (level 1); then it associates pattern1 with either an event or another first level pattern such as pattern1 to produce pattern2 (level 2), then it continues recursively to the next higher level as long as it finds events or patterns to form a yet more complex pattern. In their graphical scheme, Figures 5–7 represent the same pattern hierarchy found in the form of bracketing in the examples (1) through (3), with the vertical axis standing for the relative time of the constituting sub-patterns, whereas the horizontal axis standing for the absolute time of the recording. The vertical connected lines show that the complex patterns in (3) and (4) only occur three times each, whereas their sub-patterns have more frequent occurrences. The numbers in the boxes stand for Theme-internal IDs of patterns, whereas green boxes indicate a sub-pattern to have the function of a marker in relation to the next connected sub-pattern. As such, in the case of Figure 5 [also: example (1)] the first half of the complex pattern made of certain emotional, attitudinal and conversational events serves as a marker statistically predicting the occurrences of its second half made of certain prosodic events.

## 10 Analysis of representative sequences in ELAN

**Figure 8:**
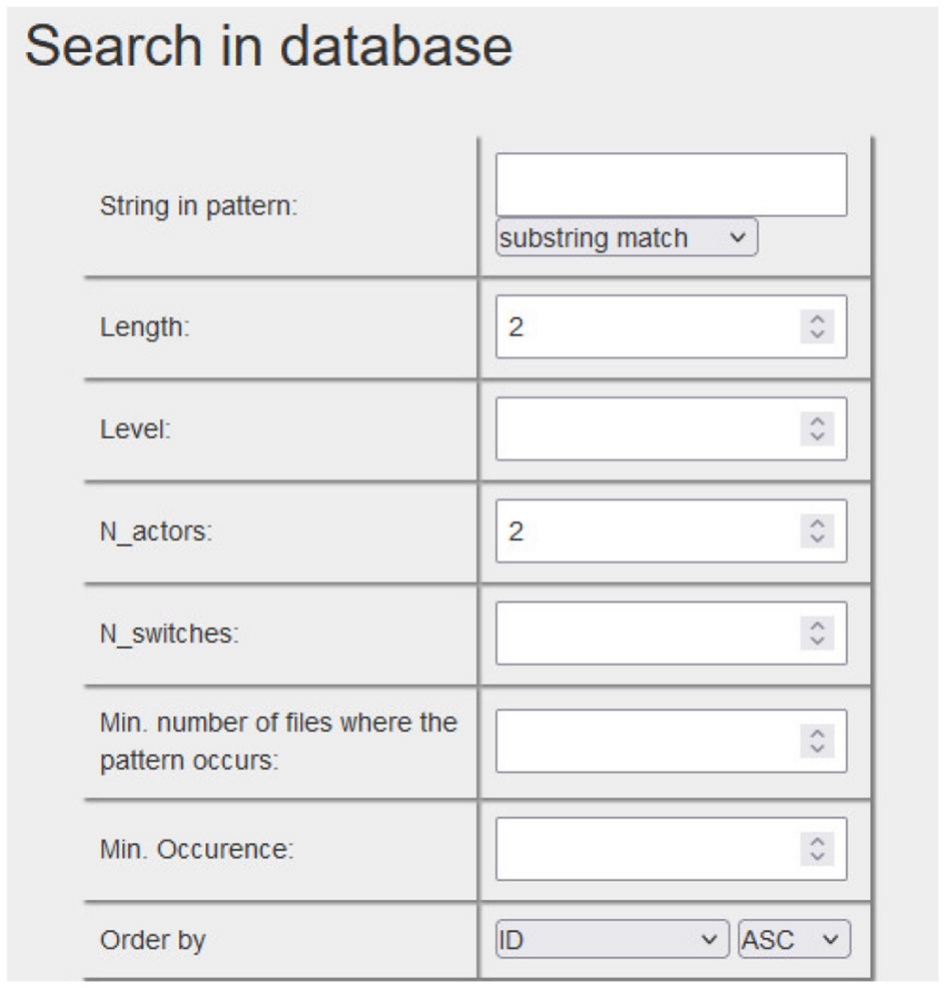
Search in a database of T-patterns and export into ELAN. Through a browser based search interface we can search for patterns according to various criteria: length = number of event types, level = number of hierarchical levels, *N*_actors = number of different actors (or other classes) in the pattern, *N*_switches = number of switches between different actors (or other classes) in the pattern, Min. number of files where the same pattern occurs, Min. occurrence of a pattern across all files.

The temporal patterns formed by different types of events that occur over time (textual, paralinguistic, kinesic, or pragmatic annotations, as well as the coding system applied to the interventions of the client and the professional) will be able to be recovered, and in their case, explored in depth qualitatively thanks to the creation (Szekrényes, 2019) of a database that allows exchange between Theme and ELAN formats (see Figure 8).

Finally, we can recover the conversation sequences of the different examples of temporal patterns to explore them with ELAN, incorporating them as a new Tier in the original ELAN file to review them in the context of the recording of the session and the rest of the annotations made in it.

**Figure 9:**
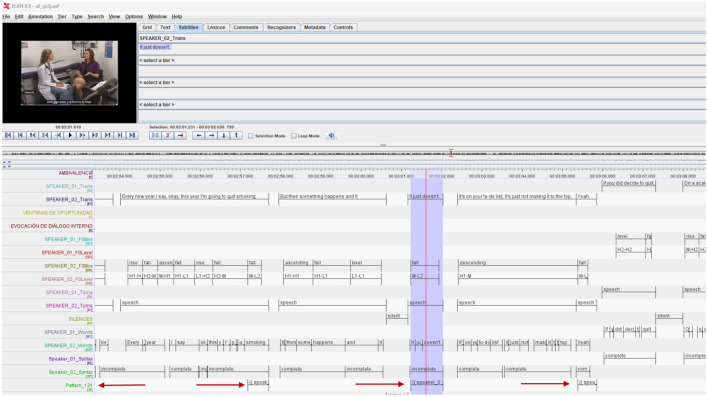
New Tier incorporated into the ELAN source file of the temporary pattern identified using Theme. Video thumbnail from “The Effective Physician: Motivational Interviewing Demonstration”, YouTube, uploaded by MerloLab, Nov 26, 2009.

As we can see in Figure 9, from the searches for temporal patterns, we have selected certain patterns that were significant and included incomplete sentences, as defined in the HuComTech project, and we have been able to open the examples of that pattern in a new Tier created in Elan, (in the image below “Pattern_131”) that we can review again in the context of the rest of the annotations and the video with the session in which the temporal pattern is found. As we can see indicated by the red arrows, annotations have been generated in Elan in the recording that exemplify the temporal pattern detected with Theme, and which can be reviewed and analyzed qualitatively taking into account their context with the rest of the annotations.

## 11 Discussion

In this article we have presented a proposal for the creation of a technological infrastructure that serves as a basis for the implementation of a therapeutic communication laboratory that assumes a mixed methods perspective that combines methodological rigor and the facilitation of collaboration between psychotherapy practitioners, researchers, and theoretical intervention models.

The incorporation of Theme (temporal pattern analysis algorithms) in Qual-Quant-Quan processes is especially important given the sequential nature of the conversation, which is the object of observation and analysis of the intervention in psychotherapy. The double back-and-forth movement between Elan and Theme that we have shown how it works based on an example of a motivational interview and the project HuComTech enhances this integration of qualitative and quantitative moments of analysis, mutually fertilizing each other.

It is worth highlighting the importance of the incorporation of automated transcriptions with artificial intelligence in Elan, and the incorporation of an automated procedure for labeling intonation and prosodic features, which is so important when generating different effects of meaning and constructing shared meanings in the conversation process.

The automation procedures that we have shown in the article make it possible to put into practice what we have called “reflective professional practice” based on observation. One of the most important impediments for psychotherapy professionals and, in general, helping professionals, to use this methodology of learning and professional improvement is the time spent preparing and accessing the most significant parts of the recordings. With this system, this handicap is overcome since these are semi-automated processes that facilitate the exploration of the sessions in a fast and operational way. Based on the infrastructure that we have described, we can work in collaboration with different groups of professionals to incorporate this methodology in their training, supervision and participation processes in research projects following this mixed methods perspective. In this way, we propose the generation of a knowledge base in which the needs of different groups of professionals can be easily incorporated into the labeling that we can add. Elan allows us to consider the different dimensions of multimodal communication, together with the labeling of the different modalities of professional intervention that can be added *ad hoc* after a qualitative analysis, but at the same time, can be systematized.

The automation of the annotation processes, up to now, the incorporation of orthographic and phonetic transcriptions, with the diarisation of speaking turns, as well as the temporal segmentation of words, allows new developments for the automatic addition of labels referring to the textual component. Thus, for example, we will be able to automatically label the terms that fall into the semantic domain of what in motivational interviewing has been related to the language of change: Desire, Ability, Reasons and Need. With the advances in computational linguistics, we can add the semantic domains of the different forms of discourse of change to automatically label them and analyze them in the temporal patterns in which they are distributed.

A wide range of possibilities for use is opened up by using this methodology:

Firstly, to advance in the automation of the automated labeling and the integration of the different multimodal components (textual, phonetic, or gestural) components function in the generation of meaning effects of therapeutic communication: formulations, the power of questions, and speaking turns in the context of the labeling of conversational sequences that are broader than adjacency pairs.

Secondly, to generate *ad hoc* annotation systems created by the professionals themselves based on their own problems and needs that arise in their daily practice, which, on the one hand, make use of the facilities of automated labeling that, with artificial intelligence, we can incorporate into observation instruments, to which they can add their own *ad hoc* annotations.

A wide range of possibilities opens up by implementing the methodology we propose. In the case of motivational interviewing or other therapies such as EMDR, it can be applied to the coding and evaluation systems of the application of the model as well as the identification of the different modalities of the discourse of change on the part of the client, and it can be analyzed from this interactional and mixed methods perspective, being useful not only to advance the proposals of the theoretical model but also in the learning, teaching, and supervision methodologies of the professionals who use it.

## Data Availability

The data analyzed in this study is subject to the following licenses/restrictions. The article is not a research paper, focus is on methods and merging available research tools. Requests to access these datasets should be directed to info@pacomolinero.net.

## Footnotes

^1^“In an equally scientific and modern spirit, another doctor, a certain Salvador Minuchin, drilled another hole in another wall of another city...In a time not very distant and in a place very far in many ways from Vienna, from Palo Alto and from Philadelphia, we looked through the mirror and were amazed at what we saw...We saw a therapist talking to his patient” (de Shazer, 1999).

^2^ https://archive.mpi.nl/tla/elan

^3^ https://patternvision.com/

^4^ https://rgai.inf.u-szeged.hu/magyarlanc

^5^ https://github.com/opencog/link-grammar

^6^ https://www.link.cs.cmu.edu/link/submit-sentence-4.html

^7^ https://www.clarin.eu/showcase/webmaus-automatic-segmentation-and-labelling-speech-signals-over-web

^8^The tool is available within the framework of the e-magyar project under the name emPros: https://e-magyar.hu/en/speechmodules/empros.

^9^ https://motivationalinterviewing.org/sites/default/files/miti42.pdf

^10^Reflections “are always offered in response to something the person has said and basically capture and return to the person something they just said. Simple reflections add little or no meaning or emphasis beyond what is said. They repeat the content, perhaps with some rephrasing, but they do not advance assumptions about what the person means. In contrast, complex reflections make a conjecture about the possible meaning beyond what is said” (Miller and Rollnick, 2015, p.560).

^11^ https://huggingface.co/pyannote/speaker-diarization-3.1

^12^ https://huggingface.co/openai/whisper-large-v3

^13^ https://github.com/szekrenyesi/prosotool

## In memoriam

We would like to dedicate this manuscript to our loving friend and coauthor Pr. M. Teresa Anguera, who passed away on January 19th, 2025. She was not only a remarkable professional but also a source of inspiration and guidance to so many of us. Her wisdom, kindness and dedication left an indelible mark on the broader research community. We have lost not just a colleague but a friend, mentor, and role model who will be deeply missed by all who knew them. Teresa impact will not be forgotten, and her legacy will live on in the work we do and the memories we hold dear.
