# Corrigendum: Therapeutic communication laboratory: integrating mixed methods with digital tools and reflective professional practice

**DOI:** 10.3389/fpsyg.2025.1632227

**Published:** 2025-06-04

**Authors:** Francisco Molinero, Gudberg K. Jonsson, M. Teresa Anguera, Laszlo Hunyadi, István Szekrényes

**Affiliations:** ^1^Mimesis, Institute for Psychosocial Studies and Analysis of Therapeutic Communication, Barcelona, Spain; ^2^Social Science Research Institute and Human Behavior Laboratory, University of Iceland, Reykjavík, Iceland; ^3^Faculty of Psychology, Institute of Neurosciences, University of Barcelona, Barcelona, Spain; ^4^Language Technology Research Group, Hungarian Research Centre for Linguistics, Budapest, Hungary; ^5^Institute of Philosophy, Faculty of Humanities, University of Debrecen, Debrecen, Hungary; ^6^Department of Digital Humanities, Faculty of Humanities, Eötvös Lóránd University, Budapest, Hungary

**Keywords:** therapeutic communication laboratory, conversational analysis in psychotherapy, therapeutic communication, analysis of temporal patterns, mixed methods, observational methodology, motivational interviewing, discourse of change

In the published article, there was an error in the funding statement. The funding statement was incomplete. The ‘National Laboratory for Digital Heritage' and the ‘Ministry of Culture and Innovation of Hungary' were not previously listed. The corrected Funding statement appears below.

